# Psychological Factors Explaining the COVID-19 Pandemic Impact on Mental Health: The Role of Meaning, Beliefs, and Perceptions of Vulnerability and Mortality

**DOI:** 10.3390/bs13020162

**Published:** 2023-02-13

**Authors:** Attà Negri, Federica Conte, Cristina L. Caldiroli, Robert A. Neimeyer, Marco Castiglioni

**Affiliations:** 1 Department of Human and Social Sciences, University of Bergamo, 24129 Bergamo, Italy; 2 Department of Psychology, University of Milano Bicocca, 20126 Milan, Italy; 3 Department of Human Sciences “R. Massa”, University of Milano Bicocca, 20126 Milan, Italy; 4 Portland Institute for Loss and Transition, Portland, OR 97223, USA

**Keywords:** meaning making, core belief violation, mortality, vulnerability, psychological processes, COVID-19 pandemic distress, mental health in COVID-19 pandemic, psychological interventions in COVID-19 pandemic

## Abstract

This study tested an expanded version of the explanatory model of the negative impact of the COVID-19 pandemic on mental health proposed by Milman and colleagues. Participants (N = 680) completed an online survey on demographic variables associated with poor pandemic mental health, COVID-19 stressors, mental health symptoms, and pandemic-related psychological processes we hypothesized as mediating mechanisms explaining the negative mental health effects of the COVID-19 stressors. Results indicated that these psychological processes (core belief violation, meaning made of the pandemic, vulnerability, and mortality perception) explained the severity of mental health symptoms to a far greater extent than COVID-19 stressors and demographics combined. In addition, these psychological processes mediated the impact of COVID-19 stressors on all mental health outcomes. Specifically, COVID-19 stressors were associated with increased core belief violation, decreased meaning making, and more intense perceived vulnerability and mortality. In turn, those whose core beliefs were more violated by the pandemic, who made less meaning of the pandemic, and who perceived a more pronounced vulnerability and mortality experienced a worse mental health condition. This study’s results suggest some possible ways of intervention in pandemic-like events useful for limiting such impact at the individual, group, social and political levels.

## 1. Introduction

Since the inception of COVID-19, many studies have investigated the multiple negative impacts of the pandemic on mental health, at the individual level, at the family level, and at the broader societal level [1,2,3,4,5,6]. Correlations have been found between different aspects of the pandemic and increased levels of depression, anxiety, self-harm, suicidal ideation, alcohol abuse, and psychophysical symptoms such as loss of appetite, dizziness, insomnia, and nausea [7,8,9,10,11].

To reduce the spread of the virus and protect public health, national and international institutions in most countries applied preventive and restrictive regulations such as physical distancing, isolation, and quarantine, with significant psychological consequences at all the levels mentioned above [12,13,14,15,16,17]. Adverse psychological effects of the restrictions include loneliness, social isolation, anger, fear, emotional reactions, and potentially harmful behaviors such as avoidance behaviors, social phobias, and substance abuse [18,19,20,21,22].

At the family level, forced cohabitation was associated with an increase in separations and divorces, e.g., [23,24], and domestic violence, e.g., [25,26,27,28]. Studies at different stages of the pandemic investigated “direct factors” (i.e., having personally contracted the virus, having severe consequences, or being in close relationship with people who died from COVID-19) and “indirect factors” (e.g., childcare loss, job loss, caregiving a disabled or ill person, socio-economic problems, working from home, distance learning, etc.). Their results have demonstrated several adverse psychological effects of the pandemic on the mental health of individuals, families, and social groups [1,18,29,30,31,32,33].

Most studies of people’s mental health during the pandemic were descriptive and reported the heavy negative impact of the phenomenon. Relatively few explanatory models have been proposed to clarify what specific psychological factors and processes are involved in the relationship between various aspects of the pandemic (including its direct and indirect stressors) and the negative mental health impact [1,29,30,32,34,35,36,37,38]. As an exception to this trend, Milman and colleagues [1] proposed an explanatory model of the relationship between pandemic-related triggers and their adverse psychological effects. The main constructs underpinning the model were two primary psychological processes derived from stress-coping theory, grief studies, and trauma research: violation of core beliefs and disrupted meaning making [39,40,41,42,43,44,45,46,47,48,49].

The theoretical rationale for the first of these processes is that individuals possess a set of core beliefs, higher-order constructs that together suggest that the world is a fair place where we can influence our circumstances and be relatively confident about the future and our ability to anticipate it. Core beliefs support the meaningfulness of our existence, our identity, our personal worth, and our relational networks [50]. Stressful or traumatic events can cause mental health disorders by violating such core beliefs, leaving individuals disoriented about who they are, their expectations for the future, and the nature of the world. According to Milman et al. [1], the COVID-19 pandemic appeared “tailor-made” to violate people’s core beliefs.

The second psychological process on which this model is based is people’s meaning making of challenging events, such as the pandemic, that force them to review or revise their violated system of core beliefs. Individuals may or may not be able to develop an understanding of a novel stressful event and integrate this understanding into their previous belief system. Disrupted meaning making implies a severe impasse in attributing meaning to the pandemic event and assimilating it into one’s worldview [51].

**Figure 1:**
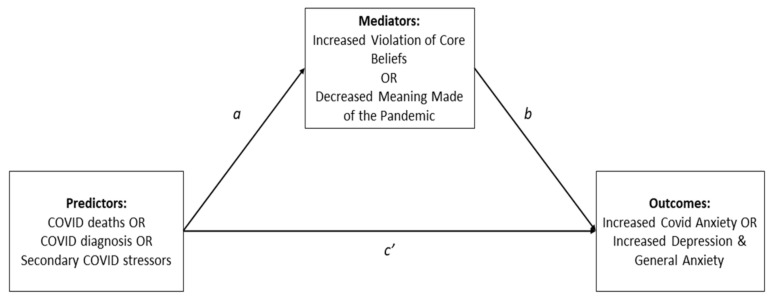
Model from Milman et al. [1]: the scheme represents the tested model of direct (*c’*) and mediated effects (*a*, *b*) of pandemic stressors (predictors) on mental health measurements (outcomes). Figure adapted here with permission.

Milman and colleagues [1] then empirically studied the role of core belief violation and disrupted meaning in regulating mental health during the pandemic. They tested the hypothesis that both psychological processes would mediate the effects of multiple pandemic stressors, accounting for adverse mental health outcomes. With a large, ethnically diverse sample from the United States, they examined the relations between demographic variables potentially associated with poor pandemic mental health (gender, age, ethnicity, education), direct COVID stressors (diagnosis, death of significant others), indirect COVID stressors (unemployment, increased living costs, loss of childcare), core belief violation, meaning attributed to the pandemic, coronavirus anxiety, depression, and general anxiety. They found that core belief violation and disrupted meaning making explained the severity of depression, general anxiety, and coronavirus anxiety (a specific kind of anxiety related to the pandemic; [20]) significantly more than demographic factors, direct COVID stressors, and indirect COVID stressors combined. Furthermore, core belief violation and disrupted meaning making significantly mediated the impact of direct COVID stressors on all mental health outcomes and completely accounted for the impact of indirect COVID stressors (See Figure 1). Their study offers an explanatory psychological model of the pandemic’s impact on mental health, suggesting that the violation of core beliefs and the inability to make meaning of the pandemic could be crucial targets for clinical intervention in the COVID context and, for those with protracted struggles in adapting, in its aftermath.

To sum up, empirical research based on this model showed that the two mediating factors (core belief violation and meaning making) explained the impact of direct and indirect pandemic-related factors on mental health outcomes (i.e., anxiety, depression, and coronavirus anxiety). On the other hand, the same model also explained how, contrary to expectations, compliance with social isolation measures did not increase the mental health burden of the pandemic but instead reduced it, apparently by reducing the impact of the pandemic on core beliefs associated with predictability and control [1]. Similarly, other investigators have found that confinement at home and the strengthening of relationships contributed, at least in well-functioning families, to reducing the violation of core beliefs and supporting functional processes of meaning making [51,52].

The present work further tested the model of Milman et al. [1] in a different context (a different country and time). Specifically, we replicated the 2020 study with the Italian population, approximately one year after the onset of the pandemic, when the “third pandemic wave” in Italy was ending and the second dose of vaccine had recently become available. Milman and colleagues [1] took a “snapshot” of the pandemic in May 2020 in the USA, while we took a snapshot in May–June 2021 in Italy (see the Supplementary Materials, Section S3, for the results of the exact replication of the analyses by Milman and colleagues). Comparing the two studies could allow us to test the robustness of the explanatory model across time and context.

As a second aim, we also sought to expand Milman et al.’s analysis [1] by considering additional factors. We added two indicators that expand and enrich their explanatory model: perceptions of being vulnerable and perceptions of one’s own mortality. Both are derived from Terror Management Theory (TMT) [53], which argues that people defend themselves against threatening events that make their mortality salient by adhering more tenaciously to a dominant cultural worldview and its prescribed attitudes and behaviors. Based on TMT, adherence to normative social prescriptions and guidelines introduced to reduce the spread of COVID-19 could reduce coronavirus anxiety [1] by offering a security-enhancing connection to the broader societal narrative of the pandemic. Thus, we hypothesized that the degree of one’s own perceived vulnerability and the salience of one’s mortality would function as additional mediators of the impact on mental health along with core belief violation and disrupted meaning making. A higher sense of vulnerability and more frequent thoughts of one’s mortality connected to the pandemic would thus lead to worse mental health outcomes.

Confirmation of this “integrative” model could help shape policy, community, and individual interventions to support mental health during the continuation of this pandemic, in its aftermath for those struggling with residual symptoms, and during similar situations in the future.

## 2. Materials and Methods

### 2.1. Participants

The study involved 680 participants (423 of whom were women) between the ages of 18 and 91 years (*M* = 52.81; *SD* = 15.94). Being at least 18 years old, speaking fluent Italian, and giving online informed consent were the only inclusion criteria. Detailed demographic information can be found in the results and in Table 1. All participants were included in the analyses. A sensitivity analysis showed that the sample allowed for reliable estimation of Pearson bivariate correlations as small as 0.11 with a statistical power of 0.80 (α = 0.05).

### 2.2. Procedure and Measures

Participants were recruited between May and June 2021 through the collaboration with four general practitioners (GPs) from Milan and Bergamo in northern Italy, two of the provinces most affected by the early outbreak of the pandemic. The GPs invited all their patients to take part in a study of the pandemic’s impact on psychological wellbeing and shared with them the link to an online set of questionnaires and questions.

All participants completed the procedure during the third pandemic wave after the harshest lockdown restrictions had been lifted. They responded to the online form at home in a single session lasting approximately 30 min. They provided their informed consent to participate after reading the study presented in the first section of the online material.

Through this procedure, we collected participants’ demographics and answers to several socio-psychological scales. The instruments were presented to all participants in the same fixed order illustrated below. Detailed questions and answer options are reported in the Supplementary Materials.

The study focused on the following variables:

**Demographics and general information**. Participants’ age, gender, nationality, education, marital status, living conditions (e.g., living with their children), caregiver status (i.e., acting as a caregiver of elderly or disabled people), occupation, and health (e.g., medical and psychological conditions predating the COVID-19 pandemic). The marital status variable was further treated to facilitate analysis: participants selected the option that best represented them among a set of four (i.e., single, in a relationship but not cohabiting, in a relationship and cohabiting, married, divorced or widowed) and their answer was then coded in four dummy variables, each representing one of the options (i.e., single “no/yes”, in a relationship but not cohabiting “no/yes”, etc.).

**Indirect COVID-19 stressors.** Conditions associated with the pandemic or with the public response to it, such as loss or reduction of employment, working with COVID patients, having to work from home or to leave the house for work even during lockdown, increased cost of living, childcare loss, and confinement measures.

**Direct COVID-19 stressors.** Being tested positive for COVID-19 and deaths of family members, friends, or acquaintances due to COVID-19. 

**Psychological factors.** Psychological variables that, according to our hypothesis, play a part in shaping the mental health outcomes of the pandemic:

- *Core Belief Inventory* (CBI) [39]. A brief measure of the effect of stressful experiences on an individual’s assumptive world or, in other words, a measure of *violation of core beliefs*. The CBI consists of nine items focusing on religious and spiritual beliefs, human nature, relationships with other people, meaning of life, and personal strengths and weaknesses. The respondents indicate the extent to which the stressful experience (in our case, the pandemic) led them to seriously examine their core beliefs. Responses are given on a six-point scale ranging from “not at all” (0) to “to a very great degree” (5). The total score is computed as the mean of individual item scores, and higher values indicate a greater degree of core beliefs violation. In this study, CBI showed excellent internal consistency (α = 0.90);
- *Integration of Stressful Life Experiences Scale—Short Form* (ISLES-SF) [54]. A brief measure of an individual’s ability to make sense of stressful events. It assesses the degree of disrupted meaning making following a specific event, in the present case, the pandemic. The ISLES-SF consists of six items, and participants express their degree of agreement on a five-point scale ranging from “strongly agree” (1) to “strongly disagree” (5). The total score is computed as the sum of item scores, and lower values indicate a greater disruption in meaning making ability. In this study, ISLES-SF showed good internal consistency (α = 0.88);
- *Vulnerability*. Participants reported their agreement with the statement “This pandemic made me feel vulnerable and fragile” using a 6-point scale (from 0 = “not at all” to 5 = “to a very high degree”);
- *Mortality.* Participants reported their agreement with the statement, “This pandemic made me think more about my own death” (from 0 = “not at all” to 5 = “to a very high degree”).

**Mental health and distress measures.** We administered a broad set of tools to investigate the impact of the pandemic on participants’ mental health.

- *Four-item Patient Health Questionnaire* (PHQ-4) [55]. An ultra-brief measure (four items) of anxiety and depression problems experienced over the last two weeks. Respondents indicate the frequency of the proposed feelings on a 4-point scale ranging from “not at all” (0) to “nearly every day” (3). The total score is determined as the sum of item scores and indicates the severity of reported symptoms. In this study, PHQ-4 showed good internal consistency (α = 0.86);
- *Coronavirus Anxiety Scale* (CAS) [20]. A brief mental health screener (five items) designed to quickly and accurately identify individuals functionally impaired by their fear and anxiety over the coronavirus. Respondents reported the frequency of given situations on a 4-point scale ranging from “not at all” (0) to “nearly every day over the last two weeks” (3). The total score is computed as the sum of item scores and indicates the degree of coronavirus dysfunctional anxiety experienced over the last two weeks. In this study, CAS showed suitable internal consistency (α = 0.86);
- *General Population—Clinical Outcomes in Routine Evaluation* (GP-CORE) [56]. A non-clinical 14-item self-report measure of wellbeing, psychological problems, and functioning for the general population (GP), derived from the Clinical Outcomes in Routine Evaluation-Outcome Measure (CORE-OM). For each item, the respondents rate themselves with reference to the last week on a 5-point scale ranging from “not at all” (0) to “most or all of the time” (4). A single overall score is created by calculating the mean score over the 14 items, where higher values indicate lower degrees of wellbeing. In this study, GP-CORE showed good internal consistency (α = 0.83);
- *Profile Of Mood States* (POMS) [57]. A self-report mood adjective checklist in which each adjective is scored from 0 (absent) to 4 (very much) based on how well each item describes the respondent’s mood during the previous week. The 58 items are grouped into six subscales: tension, anger, fatigue, depression, confusion, and vigor. An overall measure of respondents’ mood can be calculated by subtracting the vigor score from the sum of the other scales’ scores. In this study, POMS showed excellent internal consistency (α = 0.98).

### 2.3. Statistical Analyses 

**Multiple regressions.** We ran four separate multiple regressions to assess the impact of demographic characteristics, indirect and direct COVID stressors, and psychological factors (predictors) on each of the post-pandemic wellbeing measures (outcomes). Predictors were entered in a hierarchical blockwise fashion. This allowed us to test the effects of each individual predictor as well as to evaluate how different sets of predictors contributed to explaining outcome variability.

In each of the five regression analyses, we entered predictors in four blocks: (1) demographic variables (age, gender, education, marital status, number of cohabiting children, caretaker status, and pre-existing medical and psychological conditions); (2) indirect COVID stressors (loss of employment, loss of childcare, increased cost of living, working with COVID patients, forced working conditions in or outside the house, confinement); (3) direct COVID stressors (COVID diagnoses and acquaintances’ COVID-related death); (4) psychological factors (i.e., core belief violation, meaning making, vulnerability and mortality). We determined the order of the blocks based on the theoretical considerations and predictions partly derived from Milman et al. [1]. 

**Multiple mediation analyses**. We used multiple mediation analyses to test whether our data were compatible with the hypothesis that psychological factors mediate the effects of direct and indirect stressors on post-pandemic mental health. Specifically, we were interested in quantifying the indirect effects of pandemic stressors (i.e., the combination of their impact on a given mediator and that mediator’s subsequent effect on the outcome). Additionally, we quantified the stressors’ controlled direct effects; that is, their impact on the outcome keeping all potential mediators at a constant value. To do this, we implemented a regression-based approach to analyze the effects of several mediators acting simultaneously in parallel.

**Figure 2:**
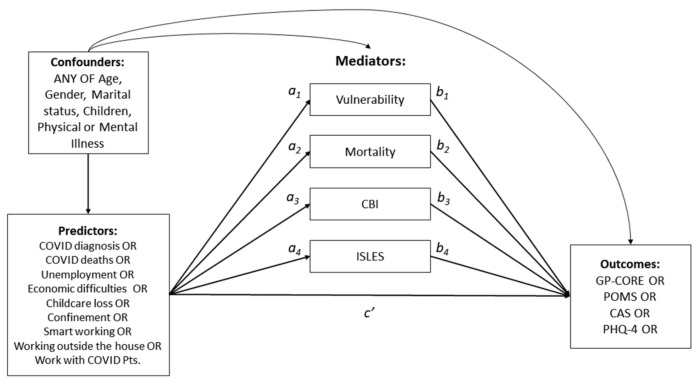
Schematic representation of the multiple mediation models. We fit a separate model for each predictor-outcome pair sharing a significant bivariate correlation. The paths marked *a_1–4_* represent the effects of each predictor on each of the four mediators. The paths marked *b_1–4_* represent the effects of each of the four mediators on each outcome variable. The path marked *c’* represents the controlled direct effect of each predictor on each outcome. All effects were estimated by conditioning on relevant confounders.

We developed a model for each predictor-outcome pair sharing a significant bivariate correlation and included in it four potential mediators (i.e., CBI, ISLES-SF, Vulnerability and Mortality perceptions) and all potential confounders. A confounder was identified as a variable that predates pandemic stressors and that is associated with both stressor and mediators, or stressor and outcome, or mediators and outcome. Examples of such variables are age, gender, or pre-existing pathologies. Each model included the controlled direct effect of the predictor on the outcome (Figure 2, *c’*) and four indirect effects (Figure 2, *a_1–4_*–*b_1–4_*), all estimated while statistically controlling for potential confounders. Indirect effects, their standard errors, and confidence intervals were estimated based on 5000 bootstrap samples.

All analyses were run in the R environment using the base package for multiple regressions [58] and the Psych package for multiple mediations [59].

## 3. Results

### 3.1. Descriptive Statistics

The respondents (N = 680) completed all questionnaires and questions (see Table 1); most of them had a bachelor’s degree or higher qualification (57.0%); the prevalent family arrangement was living with a partner (69.6%) and not cohabiting with children (60.9%). A subset of 51 individuals (7.5%) declared they were taking care of an elderly or disabled person. Approximately half of the participants had pre-existing physical illnesses (44.6%), whereas 1.9% had a pre-existing mental illness.

Almost all the respondents (93.7%) reported experiencing confinement, 68.9% reported working from home, and 59.7% leaving the house for work during lockdown (although these percentages appear contradictory, note that the questionnaire referred to the entire pandemic period and that the same person might have experienced both situations during that time). About half the sample declared that they experienced job loss or reduction (49.4%) and economic difficulties (53.1%) and were affected by the loss of childcare services (49.3%).

Only 12.5% of respondents had tested positive for COVID-19, 60.4% had had an acquaintance who died of COVID-19, and 19.8% had had a significant person who died of COVID-19.

Descriptive statistics of scores on pandemic-related psychological factors and mental health measures are reported in Table 2. The bivariate correlations among all the variables involved in the study are reported in Appendix A, Table A1.

### 3.2. Multiple Regressions

Blockwise multiple regressions (see Table 3) showed that demographic variables explained, on average, 13.3% of outcome variability. Taking into account the indirect COVID-19 stressors significantly increased the model fit when predicting mood states (POMS), whereas direct COVID-19 stressors significantly improved the COVID anxiety (CAS) model. In the GP-CORE model, the stepwise addition of indirect COVID-19 stressors first and direct COVID-19 stressors next did not lead to significant improvements in model fit. However, post hoc analyses have shown that the GP-CORE Model 3 explains significantly more variance compared to Model 1. This means that although the individual contributions of indirect and direct stressors are small, when taken together, they represent a meaningful addition to the demographic variables. Finally, psychological factors, when added to the model, provided a significant increase in the model fit in all cases. The full models, including all four blocks of predictors, explain, on average, 31.2% of outcome variability. See Table 3 to compare *R*^2^ at different steps of the blockwise regressions.

Results from the multiple regressions (see Table 4) indicated that being younger, suffering from a pre-existing mental or physical illness, and experiencing greater core belief violation and vulnerability during the pandemic consistently predicted worse post-pandemic outcomes, as assessed by the PHQ-4, GP-CORE, and POMS. In addition, economic difficulties predicted higher scores on the GP-CORE and the POMS. The CAS measure showed a slightly different pattern of results, with greater coronavirus anxiety predicted for women suffering from pre-existing mental illnesses, being exposed to COVID-related deaths, experiencing more core belief violation (CBI), or mortality perception. We also observed that those who had to leave home to work during lockdown reported less coronavirus anxiety (i.e., lower CAS scores) and that job loss or reduction was the only experience to predict better—rather than worse—scores on the PHQ-4, GP-CORE, and POMS.

### 3.3. Multiple Mediation

We fit 12 multiple mediation models for any predictor-outcome pair that showed significant bivariate correlations (see Table A1 in Appendix A). 

Table 5 reports our effects of interest: controlled direct and natural indirect effects, estimated while controlling for potential measured confounders:

- Violation of core beliefs (CBI) mediated the effects of several indirect COVID-19 stressors on post-pandemic mental health levels. It mediated the effects of unemployment and childcare loss on CAS and POMS, the effects of leaving home for work on POMS, the effects of exposure to COVID-related deaths on CAS, and the effects of economic difficulties on all four outcomes;
- Perceived vulnerability mediated the effects of economic difficulties on PHQ-4, GP-CORE, and POMS but not on CAS;
- Perception of mortality mediated the effects of one direct COVID-19 stressor (exposure to COVID-related deaths) on coronavirus anxiety level (CAS);
- Disrupted meaning making (ISLES-SF) about the pandemic did not significantly mediate the effects of any predictor on the outcomes;
- Exposure to COVID-related deaths was the only predictor to have a significant direct effect on one of the post-pandemic mental health measures, the coronavirus anxiety level (CAS).

These results are independent of participants’ gender, age, marital status, number of children living at home, and physical and mental illness (see Supplementary Results Section S2 for a list of confounders included in each model and estimates of predictor-mediator and mediator-outcome associations).

## 4. Discussion

Since the pandemic outbreak, numerous empirical studies have been published describing and demonstrating the worldwide impact of COVID-19-related events on mental health [10,60,61]. Far fewer studies, however, have proposed and tested models of psychological processes that appear to regulate the influence of the pandemic on mental health, e.g., [1,17,22,32,51,62]. Such modeling is useful and necessary to make sense of research findings and to offer operational guidance to mental health professionals on how and where to intervene to limit the negative effects of the pandemic and, if possible, turn them into possibilities for growth.

The model proposed by Milman et al. [1] was one of the first attempts to explain the psychological processes underlying the pandemic’s impact on mental health, and it received robust empirical validation by the authors investigating a U.S. population in the early months of the pandemic (May 2020). The present study is further validation of that model in a different time (May and June 2021) and country (Italy). Additional variables and a more multidimensional assessment of mental health complement the model. Specifically, as in the pioneering work of Milman et al. [1], the psychological processes explained the severity of mental health symptoms to a far greater extent than direct and indirect COVID stressors and demographics combined. In our study, to make the model more comprehensive and cover additional factors with a potential impact on mental health, we added demographic variables (marital status, living with children, the role of caregiver, previous mental or physical illness, beyond gender, age, and education, which were already considered in the original study), additional COVID-19 indirect stressors (forced working conditions—from home or outside the home, working with COVID-19 patients, confinement measures in addition to unemployment, loss of child care, and increased living costs already considered in the original study), two mediating psychological processes (awareness of mortality and perception of personal vulnerability in addition to those of disrupted meaning making and violation of core beliefs already considered in the original model), and two multidimensional mental health assessments (GP-CORE and POMS, in addition to the CAS and PHQ-4 used in the original study). 

Also, in this new comprehensive model, the psychological processes (core belief violation, disrupted meaning making, perception of vulnerability, and mortality) accounted for nearly 17% of the variance in all measures of mental health (see Table 3), which is more than two times the amount of variance explained collectively by demographics (gender, age, ethnicity, education, marital status, living with children, role of caregiver, and previous mental or physical illness), direct COVID-19 stressors (diagnosis and death), and indirect COVID-19 stressors (unemployment, loss of childcare, increased living costs, forced working conditions—from home or outside the home, working with COVID-19 patients, and confinement measures).

These findings highlight the crucial role played by attributing meaning to events that otherwise have a negative and traumatic impact on an individual’s mental health. This is even more evident if we consider that when core belief violation, disrupted meaning making and perception of vulnerability and mortality were accounted for, experiencing a COVID-19 death no longer significantly predicted decreased mental wellbeing (see Table 4). Only the anxiety about being contaminated with coronavirus is directly predicted by experiencing COVID-19 deaths among the significant relational figures, a very narrow and specific part of the discomfort experienced by the individual. Thus, the results, in accordance with Milman et al.’s study [1], highlighted the crucial mediating role of these psychological processes in linking pandemic stressors with the severity of individuals’ distress. Specifically, both direct and indirect COVID-19 stressors were associated with increased core belief violation, decreased meaning making, and more intense perceived vulnerability and mortality. In turn, those whose core beliefs were more violated by the pandemic, who made less meaning of the pandemic, and who perceived a more pronounced vulnerability and mortality experienced a worse mental health condition. In other words, the psychological processes we accounted for explained how the pandemic stressors can be associated with worse mental health. 

Going into the specifics of each psychological process investigated as a mediator of the impact of the pandemic on mental health, we can say that the pandemic represented an experience with traumatic characteristics insofar as it led to a violation of the core beliefs that individuals had about themselves, the world, and the future, placing them in a condition of compromised control and predictability. In fact, the assumptive world theory of trauma posits that if an event undermines the largely implicit core beliefs that govern everyday functioning, it takes on a traumatic quality [39,43,47,48,63]. In this sense, the pandemic and related events had a negative impact on mental health not because they were objectively traumatic but only to the extent that they assumed an existentially threatening nature. In particular, job loss or reduction, childcare loss, economic difficulties, forced work conditions (working from home or leaving home to work), and COVID-19 deaths among significant relational figures may have had a negative impact on mental health because they challenged the well-established and reassuring core beliefs of people. 

The mediating role of perceived vulnerability is consistent with this same theoretical framework, albeit with a restricted scope. According to our results, economic difficulties led to a perception of increased vulnerability, which in turn correlated with increased depression and anxiety (as assessed by PHQ-4) and psychological distress (as assessed by GP-CORE and POMS). 

The third psychological process we investigated—the capacity for meaning making—is theoretically complementary to the previous ones: the more that individuals can make sense of events, including traumatic ones, the weaker the negative impact these events have on mental health [46,63,64]. In our study, the inability to make meaning of the pandemic did not significantly mediate stressor-outcome associations. The limited role played by meaning making in our model compared to that of Milman et al. [1] could be explained by the fact that the instrument used to measure it (ISLES) is designed to evaluate the acute impact of discrete events (such as a bereavement or physical trauma) rather than prolonged and pervasive experiences such as a pandemic. On the other hand, the finding that having a mental or physical illness predating the pandemic and being younger predicts poorer mental health seems to be consistent with our theory. Young age and illnesses may make the process of integrating and reconstructing the meaning of negative and unexpected events less efficient.

The final moderating psychological process we added to extend the model was the perception of one’s own mortality. According to Terror Management Theory (TMT) [53], humans are probably the only ones who are aware of their own mortality, unlike all other living beings. However, the thought of one’s own mortality and death is one of the most difficult mental health challenges, so humans have developed several defenses against distress arising from events or feelings that bring them closer to the idea of their own death. One of these is to adhere more tenaciously to a dominant cultural worldview and its prescribed attitudes and behaviors to feel that they belong to a larger entity that will survive their own death. According to TMT, however, death anxiety, precisely because it is psychically intolerable, is mostly managed at the unconscious level. If the perception of one’s own death and mortality enters the focus of conscious attention, defenses against it have failed, or alternatively, but more rarely, there has been an effort to integrate one’s death into a larger value perspective. The question we asked participants about how close the pandemic had brought them to the idea of their own death thus concerns the level of awareness of this threat, which is usually a sign that the normal anxiety buffering against death did not work as well as they could. In this sense, the higher the mortality perception of the respondents, the worse the expected pandemic impact on mental health. This occurred only with respect to coronavirus anxiety (CAS): our results have shown that mortality perception partially mediated the negative association between the death of a significant relational figure and respondents’ coronavirus anxiety.

In sum, the results suggest that the pandemic exerted its negative influence on mental health via specific psychological processes engaged in meaning attribution to the experiences. The negative impact on mental health was directly associated with the degree to which the pandemic has violated people’s core beliefs, increased the individuals’ vulnerability and mortality perception, and challenged individuals’ ability to make sense of events and integrate the unexpected into their world of meaning.

The counterintuitive result we found that job loss or reduction predicts better mental health could be viewed as supporting this interpretation. It could be that job loss or reduction was experienced as a relief, allowing people not to risk exposure to COVID-19 in the workplace. In addition, the increased closeness with family members and the home environment may have strengthened people’s core beliefs and activated shared processes of meaning making about the pandemic in primary relational contexts. In support of this interpretation, previous studies demonstrated that when the pandemic left core beliefs intact, people experienced lower anxiety and COVID-19 stress [38]. In addition, despite the seemingly stressful nature of social isolation, the choice to socially isolate appeared to mitigate COVID-19 anxiety by facilitating meaning making and by preserving core beliefs in the controllable and predictable nature of the world [1,32].

Limitations should be considered when interpreting our results and may help inform future research. The main ones are related to the research design and enrolment of participants. The cross-sectional instead of longitudinal design does not allow the exclusion of alternative causal orders in the hypothesized relations: poor mental health could disrupt core beliefs, heighten perceptions of vulnerability and mortality, and hinder meaning making. Alternatively, mental health and other psychological processes could be directly shaped by pandemic exposures and then mutually exacerbate one another over time. However, as Milman et al. [31] state, there is already research “highlighting core belief violation and disrupted meaning making as processes driving poor mental health following stressful events [44,45,46,63,64]”. Thus, “the plausibility of these various alternatives must be assessed using prospective longitudinal designs or intervention studies that address pandemic-related mental health by targeting challenged belief systems” [31]. Furthermore, given the direct relevance of results from this field of research to the design of real-world interventions, it is crucial that future studies fully employ instruments designed to improve methodological transparency and facilitate their replicability, such as (but not limited to) pre-registration and registered reports.

The second limitation concerns the representativeness of the surveyed sample, who were patients of general practitioners and responded to their request to fill out the questionnaires. Many of them had a medical condition for which they were being treated by their physicians, such that replicating the study with different populations would increase the external validity and generalizability of the results obtained.

However, we believe that the value of this research surpasses its limitations. This study represents a second confirmation of the model proposed by Milman et al. [1] on a different population, at a time more distant from the pandemic outbreak and with a larger number of multidimensional assessment tools. The proposed explanatory model, unlike many others, was properly psychological; that is, it focused on the psychic processes that theoretically lead to a worsening mental health status. The results undoubtedly need to be explored through different research designs, but they still offer a useful reference for those who must intervene in pandemic-like events to limit such impact at the individual, group, social and political levels. The most appropriate choices at all levels of intervention should aim not at nonspecific psychological containment and support but at creating contexts that can foster the creation of individual and communitarian meanings, strengthen people’s core beliefs, and reduce preoccupation with mortality and the perception of personal vulnerability. This is certainly possible in the traditional psychotherapeutic setting [50,65,66,67,68,69], but it can also involve activating primary relational, social, and cultural contexts capable of generating collective and participatory meanings for the unexpected and often traumatic events that globalized life will increasingly lead us to experience.

## Figures and Tables

**Table 1:** Participants’ distribution for demographic variables and for indirect and direct stressors.

Demographic Variables	
Education	Primary school = 33 (4.8%) Secondary school = 260 (38.2%) Post-secondary school = 387 (57.0%)
Marital status	Single = 97 (14.3%) In a relationship (not living together) = 48 (7.0%) In a relationship (living together) = 473 (69.6%) Divorced/widowed = 62 (9.1%)
Children in the house	None = 414 (60.9%) One = 109 (16.0%) Two = 127 (18.7%) Three = 26 (3.8%) Four = 4 (0.6%)
Caregiver role	51 (7.5%)
Physical illness	303 (44.6%)
Mental illness	13 (1.9%)
**Indirect COVID-19 Stressors**	
Job loss or reduction	336 (49.4%)
Economic difficulties	361 (53.1%)
Childcare loss	335 (49.3%)
Confinement	637 (93.7%)
Working from home	467 (68.7%)
Leaving home to work	406 (59.7%)
Work with COVID patients	30 (4.4%)
**Direct COVID-19 Stressors**	
COVID diagnosis	85 (12.5%)
COVID death	None = 134 (19.7%) Acquaintances = 411 (60.4%) Significant others = 135 (19.8%)

**Table 2:** Descriptive statistics for psychological factors and mental health measures.

	Range	M (SD)
Pandemic-related psychological factors		
CBI	0; 5	2.26 (1.27)
ISLES-SF	6; 30	19.08 (6.85)
Vulnerability	0; 5	2.74 (1.67)
Mortality	0; 5	2.36 (1.77)
Post-pandemic mental health measures		
PHQ	0; 12	3.97 (3.09)
CAS	0; 19	1.38 (2.71)
GP-CORE	0; 3.57	1.51 (0.6)
POMS	−29; 187	33.1 (41.35)

**Table 3:** Proportion of variance explained at each level of the blockwise multiple regression models.

Outcome	Model 1	Model 2	Model 3	Model 4
	Demographics	Demographics + Indirect stressors	Demographics + Indirect stressors + Direct stressors	Demographics + Indirect stressors + Direct stressors + Psychol. factors
CAS	*R*^2^ = 0.139	*R*^2^ = 0.144	*R*^2^ = 0.156 **	*R*^2^ = 0.288 ***
PHQ-4	*R*^2^ = 0.144	*R*^2^ = 0.145	*R*^2^ = 0.147	*R*^2^ = 0.327 ***
GP-CORE	*R*^2^ = 0.115	*R*^2^ = 0.119	*R*^2^ = 0.122 ^a^	*R*^2^ = 0.298 ***
POMS	*R*^2^ = 0.132	*R*^2^ = 0.140 *	*R*^2^ = 0.141	*R*^2^ = 0.334 ***
Mean	*R*^2^ = 0.133	*R*^2^ = 0.137	*R*^2^ = 0.142	*R*^2^ = 0.312

Note. * a significant *R*^2^ increase compared to the previous model at the following significance levels: * *p* ≤ 0.05, ** *p* ≤ 0.01, *** *p* ≤ 0.001; ^a^ post hoc comparison showed that Model 3 explained significantly more variance than Model 1; however, single-step *R*^2^ changes (from Model 1 to 2 and from Model 2 to 3) were not significant.

**Table 4:** Regression coefficients for the final models in the blockwise multiple regression analyses.

Predictors	CAS		PHQ-4		GP-CORE		POMS	
	Estimate (b)	*p*	Estimate (b)	*p*	Estimate (b)	*p*	Estimate (b)	*p*
(Intercept)	−0.33	*0.685*	**3.31**	***<0.001***	**1.35**	***<0.001***	13.90	*0.245*
Gender	**−0.40**	***0.036***	−0.31	*0.139*	0.03	*0.449*	0.95	*0.737*
Education	−0.26	*0.100*	0.16	*0.360*	0.02	*0.562*	3.53	*0.138*
Age	0.01	*0.355*	**−0.04**	***<0.001***	**−0.01**	***<0.001***	**−0.40**	***<0.001***
MS single ^a^	0.05	*0.816*	0.05	*0.849*	0.08	*0.098*	4.52	*0.171*
MS relationship ^a^	−0.15	*0.587*	0.07	*0.814*	−0.06	*0.379*	−0.88	*0.834*
MS cohabiting ^a^	−0.10	*0.554*	−0.13	*0.469*	−0.04	*0.217*	−2.37	*0.330*
Children	−0.06	*0.625*	0.11	*0.373*	−0.01	*0.819*	1.03	*0.545*
Caretaker role	0.22	*0.517*	0.74	*0.051*	0.10	*0.199*	8.69	*0.086*
Physical illness	0.18	*0.389*	**0.58**	***0.011***	**0.16**	***0.001***	**6.15**	***0.044***
Mental illness	**5.29**	***<0.001***	**4.34**	***<0.001***	**0.91**	***<0.001***	**66.32**	***<0.001***
Job loss or reduction	0.02	*0.944*	**−0.79**	***0.044***	**−0.20**	***0.011***	**−12.09**	***0.021***
Economic difficulties	0.10	*0.764*	0.67	*0.074*	**0.15**	***0.044***	**12.38**	***0.013***
Childcare loss	0.46	*0.094*	0.02	*0.950*	0.03	*0.629*	1.38	*0.732*
Working from home	−0.14	*0.579*	0.02	*0.956*	−0.02	*0.778*	−1.52	*0.678*
Leaving home to work	**−0.54**	***0.027***	−0.32	*0.232*	−0.03	*0.523*	−1.48	*0.677*
Confinement	0.40	*0.287*	−0.28	*0.500*	−0.02	*0.803*	−5.10	*0.361*
Working with COVID patients	−0.05	*0.915*	−0.21	*0.668*	−0.10	*0.284*	−0.61	*0.926*
COVID diagnosis	−0.03	*0.912*	0.02	*0.950*	0.01	*0.875*	−1.81	*0.654*
COVID death	**0.32**	***0.025***	0.04	*0.815*	0.01	*0.816*	0.51	*0.810*
CBI	**0.50**	***<0.001***	**0.47**	***<0.001***	**0.10**	***<0.001***	**7.26**	***<0.001***
ISLES	−0.02	*0.117*	−0.01	*0.530*	−0.01	*0.108*	−0.22	*0.272*
Vulnerability	0.05	*0.529*	**0.58**	***<0.001***	**0.08**	***<0.001***	**7.43**	***<0.001***
Mortality	**0.26**	***<0.001***	−0.02	*0.819*	0.02	*0.094*	−0.22	*0.822*

Note. Unstandardized regression coefficients. Bold numbers indicate significant effects *p* < 0.05; MS = marital status; ^a^ compared to marital status = “divorced or widowed”.

**Table 5:** Direct and indirect effects in multiple mediation models.

Stressor	Outcome	Mediator				Controlled Direct Effect ^b^
		CBI ^a^	ISLES ^a^	Vulnerability ^a^	Mortality ^a^	
Job loss or reduction	CAS	**0.24** **[0.11, 0.4]**	0.05 [0.00, 0.12]	0.01 [−0.02, 0.04]	0.05 [−0.02, 0.13]	0.07 *0.700*
Job loss or reduction	POMS	**3.22** **[1.44, 5.5]**	0.55 [−0.35, 1.62]	1.28 [−0.59, 3.25]	−0.02 [−0.56, 0.48]	−3.02 *0.268*
Childcare loss	CAS	**0.16** **[0.04, 0.31]**	0.06 [0.00, 0.14]	0.01 [−0.02, 0.05]	0.04 [−0.04, 0.13]	0.19 *0.341*
Childcare loss	POMS	**2.10** **[0.55, 4.18]**	0.58 [−0.43, 1.70]	1.35 [−0.80, 3.61]	−0.01 [−0.46, 0.43]	−0.65 *0.828*
Economic difficulties	CAS	**0.28** **[0.14, 0.45]**	0.05 [0.00, 0.11]	0.01 [−0.02, 0.06]	0.06 [−0.01, 0.14]	0.11 *0.544*
Economic difficulties	PHQ-4	**0.24** **[0.11, 0.39]**	0.02 [−0.05, 0.08]	**0.17** **[0.02, 0.33]**	0.00 [−0.05, 0.04]	−0.11 *0.579*
Economic difficulties	GP-CORE	**0.05** **[0.02, 0.08]**	0.01 [0.00, 0.03]	**0.02** **[0.00, 0.05]**	0.01 [0.00, 0.02]	−0.02 *0.670*
Economic difficulties	POMS	**3.61** **[1.67, 5.94]**	0.45 [−0.37, 1.43]	**2.16** **[0.28, 4.34]**	0.00 [−0.54, 0.52]	1.38 *0.615*
Working from home	PHQ-4	0.03 [−0.05, 0.13]	0.00 [−0.02, 0.02]	0.07 [−0.09, 0.25]	0.00 [−0.03, 0.03]	−0.20 *0.367*
Working from home	POMS	0.55 [−0.88, 2.18]	0.04 [−0.30, 0.46]	0.89 [−1.19, 3.13]	0.01 [−0.34, 0.38]	−2.09 *0.480*
Leaving home to work	POMS	**2.59** **[1.00, 4.67]**	0.20 [−0.22, 0.97]	1.38 [−0.53, 3.51]	−0.03 [−0.61, 0.47]	−2.28 *0.415*
COVID-19 deaths	CAS	**0.08** **[0.00, 0.17]**	−0.01 [−0.04, 0.02]	0.01 [−0.03, 0.05]	**0.10** **[0.04, 0.19]**	**0.31** ***0.029***

Note. Each model included confounders (specified in the main text) besides the stressor, mediator, and outcome variables illustrated here. ^a^ bootstrapped estimates and confidence intervals are shown; ^b^ multiple regression coefficient and *p*-value are shown. In bold, the significant effects (CIs do not include 0 or *p* < 0.05).

## Data Availability

The data presented in this study are openly available on OSF platform and available on request from the corresponding author.

## Supplementary Materials

The following supporting information can be downloaded at: https://www.mdpi.com/article/10.3390/bs13020162/s1, Note on measures collected and used; Questions concerning demographics and COVID-related stressors; Figures S1–S12: Multiple mediation model graphs with effects magnitude and significance; Tables S1–S2: Replication of Milman et al. (2020) mediation models.

## Appendix A

**Table A1:** Bivariate correlations among all the variables involved in the study.

	Age	Gender	MS Single	MS Relationship	MS Cohabiting	MS Divorced or Widowed	Children	Caretaker Role	Physical Illness	Mental Illness
Age										
Gender	0.16 ***									
MS single	−0.29 ***	−0.02								
MS relationship	−0.27 ***	0.01	−0.11 **							
MS cohabiting	0.24 ***	0.03	−0.62 ***	−0.42 ***						
MS divorced/widowed	0.21 ***	−0.04	−0.13 ***	−0.09 *	−0.48 ***					
Children	−0.11 **	−0.12 **	−0.25 ***	−0.15 ***	0.30 ***	−0.05				
Caretaker role	0.00	−0.05	0.17 ***	−0.01	−0.11 **	−0.01	−0.05			
Physical illness	0.47 ***	0.09 *	−0.06	−0.10 *	0.05	0.08 *	−0.19 ***	−0.02		
Mental illness	−0.03	−0.06	0.00	0.00	−0.02	0.03	0.01	0.04	−0.13 **	
COVID diagnosis	−0.01	−0.01	−0.08 *	−0.05	0.08 *	0.02	0.12 **	−0.04	−0.09 *	0.08 *
COVID death	−0.01	−0.05	−0.01	−0.03	0.07	−0.07	0.05	−0.02	−0.01	0.05
Job loss or reduction	−0.17 ***	−0.06	−0.04	0.11 **	−0.06	0.05	0.09 *	−0.01	−0.08 *	0.10 *
Economic difficulties	−0.12 **	−0.02	−0.03	0.06	−0.04	0.04	0.11 **	−0.03	−0.03	0.09 *
Childcare loss	−0.17 ***	−0.08 *	−0.13 ***	0.00	0.11 **	−0.02	0.44 ***	−0.02	−0.14 ***	0.08 *
Working from home	−0.29 ***	−0.09 *	0.07	0.00	−0.05	0.00	0.15 ***	0.06	−0.17 ***	0.09 *
Leaving home to work	−0.22 ***	−0.06+	0.00	0.00	0.01	−0.02	0.17 ***	−0.02	−0.16 ***	0.09 *
Working w/COVID patients	−0.08 *	0.00	0.04	0.00	−0.06	0.06	0.04	−0.01	−0.08 *	−0.03
Confinement	−0.03	−0.02	0.04	0.00	−0.05	0.04	0.00	−0.04	0.00	0.04
CBI	−0.22 ***	−0.24 ***	0.05	0.03	−0.07	0.03	0.05	−0.02	−0.08 *	0.11 **
ISLES	−0.08 *	−0.09 *	−0.01	0.08	−0.05	0.02	0.06	0.00	−0.02	0.05
Vulnerability	−0.16 ***	−0.21 ***	0.02	0.04	−0.06	0.04	0.06	0.00	−0.07	0.12 **
Mortality	0.03	−0.15 ***	−0.03	−0.04	−0.02	0.10 **	0.08 *	0.03	0.02	0.08
CAS	0.00	−0.17 ***	0.00	−0.02	−0.04	0.08 *	0.03	0.04	0.01	0.33 ***
PHQ4	−0.26 ***	−0.20 ***	0.09 *	0.07	−0.10 *	−0.01	0.06	0.07	−0.06	0.24 ***
GP-CORE	−0.19 ***	−0.11 **	0.13 ***	0.03	−0.12 **	0.02	0.00	0.05	0.01	0.25 ***
POMS	−0.24 ***	−0.14 ***	0.12 **	0.05	−0.11 **	−0.01	0.04	0.06	−0.07	0.27 ***
	**COVID Diagnosis**	**COVID Death**	**Job Loss Or Reduction**	**Economic Difficulties**	**Childcare Loss**	**Working from Home**	**Leaving Home to Work**	**Working with COVID Patients**	**Confinement**	
COVID diagnosis										
COVID death	0.00									
Job loss or reduction	0.08 *	0.02								
Economic difficulties	0.11 **	0.01	0.84 ***							
Childcare loss	0.12 **	−0.02	0.60 ***	0.58 ***						
Working from home	0.03	0.00	0.44 ***	0.39 ***	0.46 ***					
Leaving home to work	0.09 *	0.00	0.54 ***	0.50 ***	0.55 ***	0.51 ***				
Working with COVID patients	0.01	0.08 *	0.00	−0.01	0.00	−0.04	0.10 **			
Confinement	0.03	0.00	0.04	0.07	0.10 **	0.24 ***	0.07	−0.06		
CBI	0.11 **	0.09 *	0.23 ***	0.24 ***	0.15 ***	0.11 **	0.20 ***	0.01	0.03	
ISLES	−0.01	−0.03	0.19 ***	0.17 ***	0.19 ***	0.04	0.11 **	0.00	−0.05	
Vulnerability	0.09 *	0.11 **	0.10 *	0.11 **	0.10 *	0.10 *	0.10 **	−0.02	0.02	
Mortality	0.09 *	0.14 ***	0.07	0.07	0.07	0.00	0.07	0.00	0.02	
CAS	0.06	0.13 ***	0.11 **	0.12 **	0.10 **	0.02	0.03	−0.02	0.05	
PHQ4	0.06	0.07	0.06	0.10 **	0.07	0.09 *	0.06	−0.01	0.01	
GP-CORE	0.05	0.06	0.05	0.10 *	0.05	0.06	0.05	−0.04	0.02	
POMS	0.05	0.07	0.09 *	0.14 ***	0.09 *	0.09 *	0.10 *	0.00	0.00	
	**CBI**	**ISLES**	**Vulnerability**	**Mortality**	**CAS**	**PHQ-4**	**GP-CORE**			
CBI										
ISLES	0.29 ***									
Vulnerability	0.64 ***	0.24 ***								
Mortality	0.51 ***	0.20 ***	0.60 ***							
CAS	0.40 ***	0.19 ***	0.35 ***	0.37 ***						
PHQ4	0.45 ***	0.17 ***	0.49 ***	0.30 ***	0.46 ***					
GP-CORE	0.43 ***	0.19 ***	0.45 ***	0.33 ***	0.44 ***	0.77 ***				
POMS	0.46 ***	0.19 ***	0.49 ***	0.30 ***	0.49 ***	0.80 ***	0.84 ***			

Note. MS = marital status; * *p* < 0.05; ** *p* < 0.01; *** *p* < 0.001.
