# Supplementary material for: Psychological Factors Explaining the COVID-19 Pandemic Impact on Mental Health: The Role of Meaning, Beliefs, and Perceptions of Vulnerability and Mortality
Source: Behav Sci (Basel). 2023 Feb 13;13(2):162. doi: 10.3390/bs13020162 (PMC9952304; doi:10.3390/bs13020162)
Supplement: Supplementary file 1 [file behavsci-13-00162-s001.zip › behavsci-2169562-supplementary.pdf]

Supplementary Materials for:

Psychological factors explaining the COVID-19 pandemic impact on mental health: the role of meaning, beliefs, and perceptions of vulnerability and mortality

Attà Negri [1,*], Federica Conte [2], Cristina L. Caldiroli [3], Robert A. Neimeyer [4] and Marco Castiglioni [3]

[1] Department of Human and Social Sciences, University of Bergamo, 24129 Bergamo, Italy
[2] Department of Psychology, University of Milano Bicocca, 20126 Milan, Italy
[3] Department of Human Sciences "R. Massa", University of Milano Bicocca, 20126 Milan, Italy
[4] Portland Institute for Loss and Transition, Portland, OR 97223, USA
* Correspondence: atta.negri@unibg.it

Summary

1. Supplementary Methods 2
   1.1 Note on measures collected and used 2
   1.2 Questions concerning demographics and COVID-related stressors 3
2. Supplementary Results 5
   2.1 Multiple mediation models 5
      Predictor: Job Loss or Reduction 5
      Predictor: Childcare loss 7
      Predictor: Economic difficulties 8
      Predictor: Working from Home 10
      Predictor: Leaving Home to Work 11
      Predictor: COVID-19 Deaths 11
3. Replication of Milman et al. (2020) Mediation models 12

1. Supplementary Methods

1.1 Note on measures collected and used

The data collection protocol included measures that were not used in this study. These measures will be briefly mentioned below for sake of completeness and are currently being analysed to address different theoretical questions in separate manuscripts (in preparation). The measures included in the analyses of the present work are fully described in the main manuscript.

Data collection protocol – instruments administered in the following fixed order:

- Questions concerning demographics and COVID-related stressors
- Vulnerability: "*This pandemic made me feel vulnerable and fragile*" answered on a 6-point scale (from 0 = "*not at all*" to 5 = "*to a very high degree*")
- Mortality: "*This pandemic made me think more about my own death*" answered on a 6-point scale (from 0 = "*not at all*" to 5 = "*to a very high degree*").
- Core Belief Inventory (CBI)
- Integration of stressful life experiences scale–short form (ISLES-SF)
- Four item Patient Health Questionnaire (PHQ-4)
- Coronavirus Anxiety Scale (CAS)
- Satisfaction With Life Scale (SWLS, Diener et al., 1985)*
- Post-Traumatic Growth Inventory (PTGI, Tedeschi & Calhoun, 1996)*
- General Population - Clinical Outcomes in Routine Evaluation (GP-CORE)
- Profile Of Mood States (POMS)

* measures used to address different research questions in separate manuscripts.

1.2 Questions concerning demographics and COVID-related stressors

Participants completed the following questionnaire. Where answer options are not specified, participants were required to enter their own answer. The numbers in brackets next to answer options correspond to the value associated with that answer option in the analyses (e.g., sex was coded 0 for males and 1 for females).
Each of the stressful events listed in the last question was coded 0 if absent/ not selected and 1 if selected.

Please answer the following questions as accurately as possible:

Demographic information:

1) Age:

2) Sex:
Female (0)
Male (1)

3) Nationality:

4) Town of Residence:

5) Highest educational achievement:
Primary school (1)
Secondary school (2)
Post-secondary school (3)

6) Marital status:
Single (1)
In a relationship NOT cohabiting (2)
In a relationship and cohabiting (3)
Widowed/ Divorced (4)

8) If you have children, how many of them live with you?

7) Occupation
Student (1)
Short-term job (2)
Long-term job (3)
Unemployed and looking for job (4)
Unemployed NOT looking for job (5)
Retired (6)

9) Please list any medical and/or psychological illnesses currently affecting you:

10) Are you currently involved in psychological therapy?
No (0)
Yes (1)

11) Are you currently involved in psychopharmacological therapy?
No (0)
Yes (1)

Questions about COVID-19 (infection, illness, hospitalization) involving you and your acquaintances

1) Have you ever been diagnosed with Coronavirus?
No (0)
Yes (1)

2) Did you know anybody who died because of Coronavirus?
No (0)
Yes, but I didn't know them personally or directly (1)
Yes, someone significant in my life has died because of Coronavirus (2)

Stressful events related to the COVID-19 Pandemic
Please, answer the following questions using the scale provided below each one. Keep in mind that 1 corresponds to "not at all stressful" and 7 corresponds to "extremely stressful".

1) Did the pandemic cause you stress? *
2) How stressful was the first pandemic wave (from February to May 2020)? *
3) How stressful was the second pandemic wave (from October to December 2020)? *
4) How stressful was the second pandemic wave (from February to March 2021)? *
5) What is your current level of stress due to the pandemic? *
6) Please mark from the following list all the events that you experienced during the pandemic:
- Job loss or reduction (0/1)
- Economic difficulties (0/1)
- Loss of childcare (0/1)
- Confinement (0/1)
- Working from home (0/1)
- Leaving the house for work during lockdown periods (0/1)
- Working with COVID-19 patients (0/1)
- Other (please describe)

7) What is the MOST POSITIVE event that happened to you during the pandemic? *
8) What is the MOST NEGATIVE event that happened to you during the pandemic? *

* measures used to address different research questions in separate manuscripts.

2. Supplementary Results

2.1 Multiple mediation models

Detailed results for the 12 multiple mediation models are reported below, grouped by predictor. For each mediation model we include:

- A scheme reporting predictor – mediators and mediators - outcome associations, as well as controlled direct effect and their significance
- The list of confounders included and statistically controlled for in the model.

<u>Predictor: Job Loss or Reduction</u>

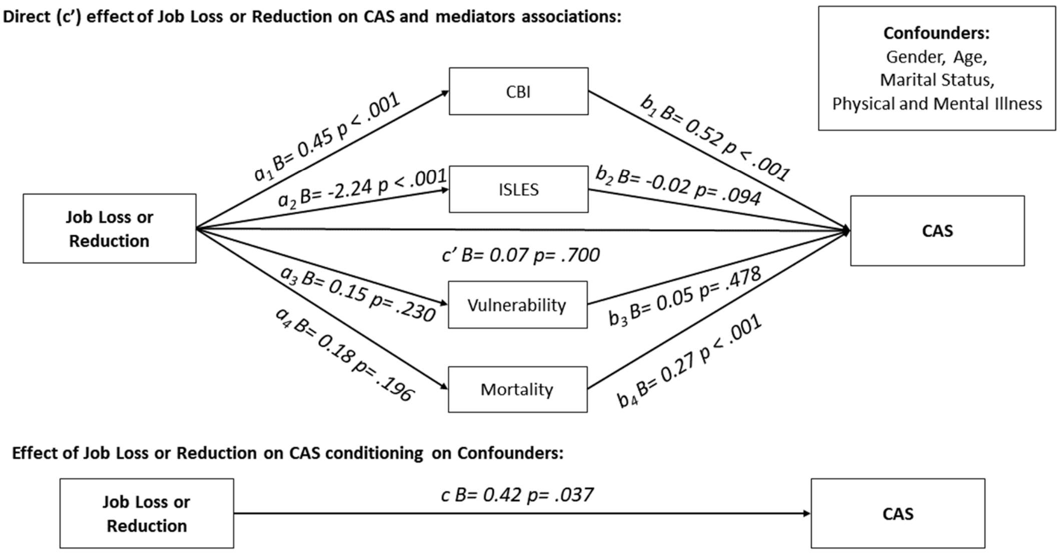

Figure S1. Mediation of the effect of Job loss or reduction on CAS.

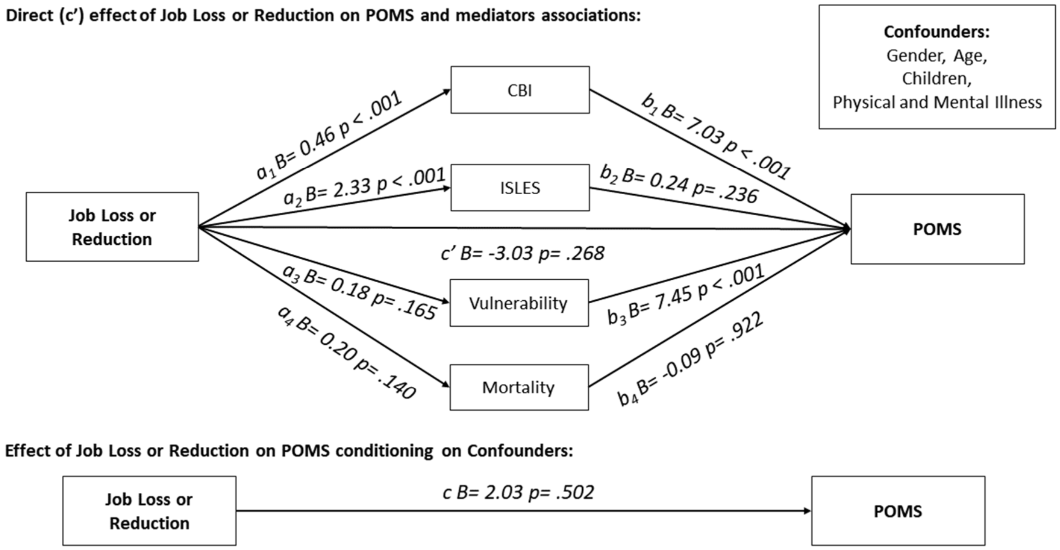

Figure S2. Mediation of the effect of Job loss or reduction on POMS.

<u>Predictor: Childcare loss</u>

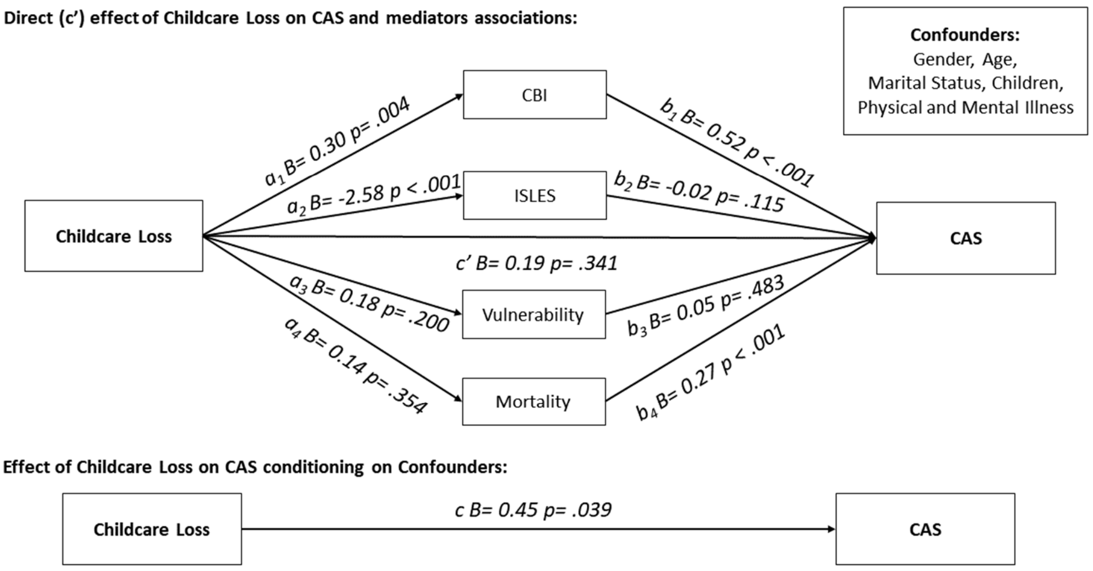

Figure S3. Mediation of the effect of Childcare loss on CAS.

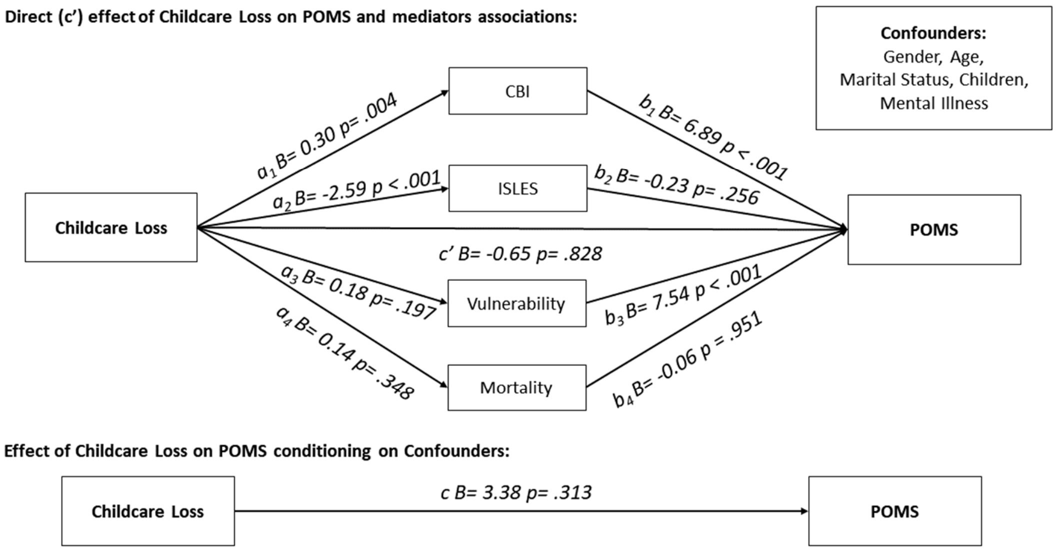

Figure S4. Mediation of the effect of Childcare loss on POMS.

Predictor: Economic difficulties

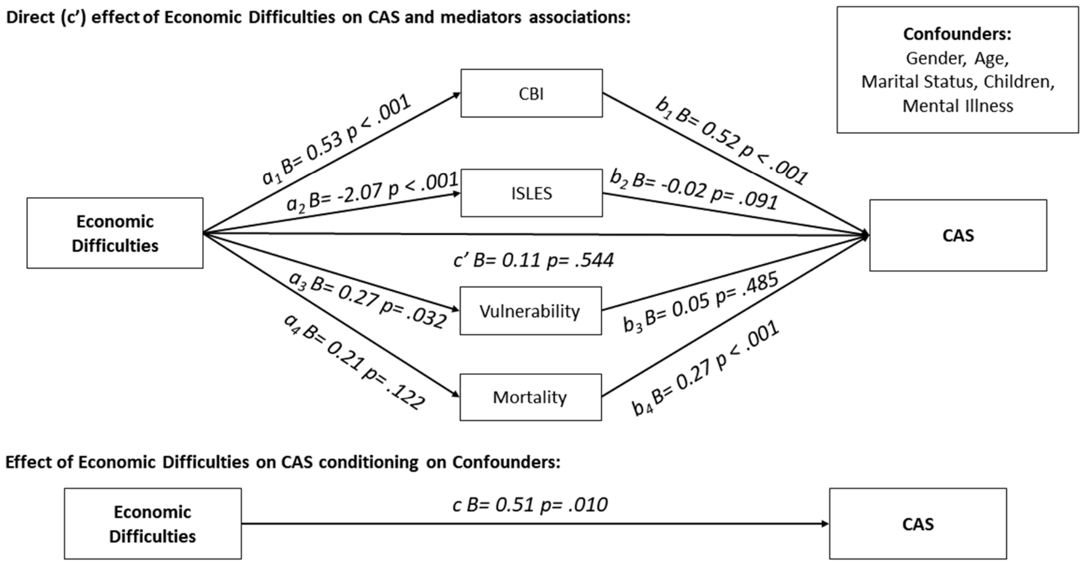

Figure S5. Mediation of the effect of Economic difficulties on CAS.

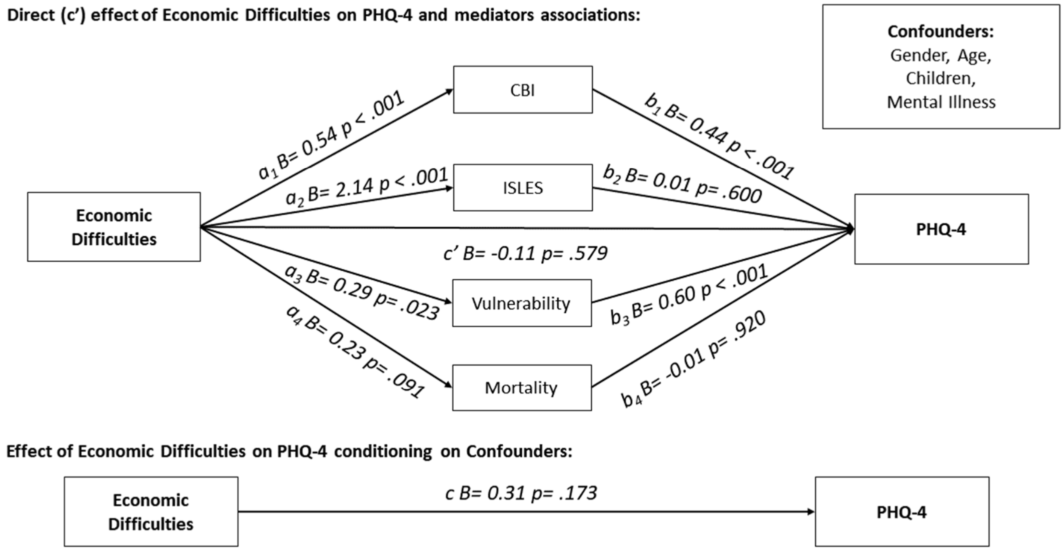

Figure S6. Mediation of the effect of Economic difficulties on PHQ-4.

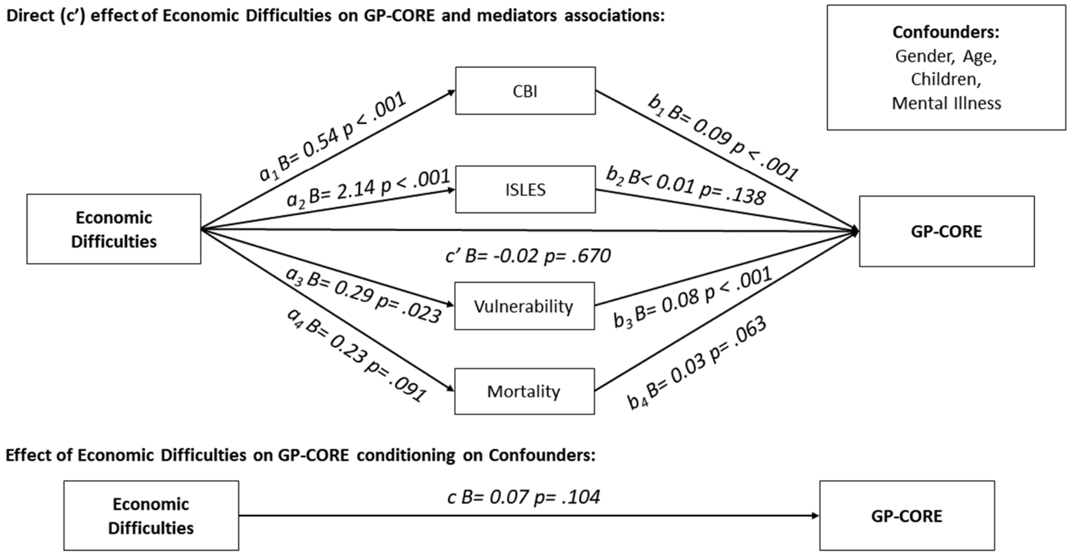

Figure S7. Mediation of the effect of Economic difficulties on GP-CORE.

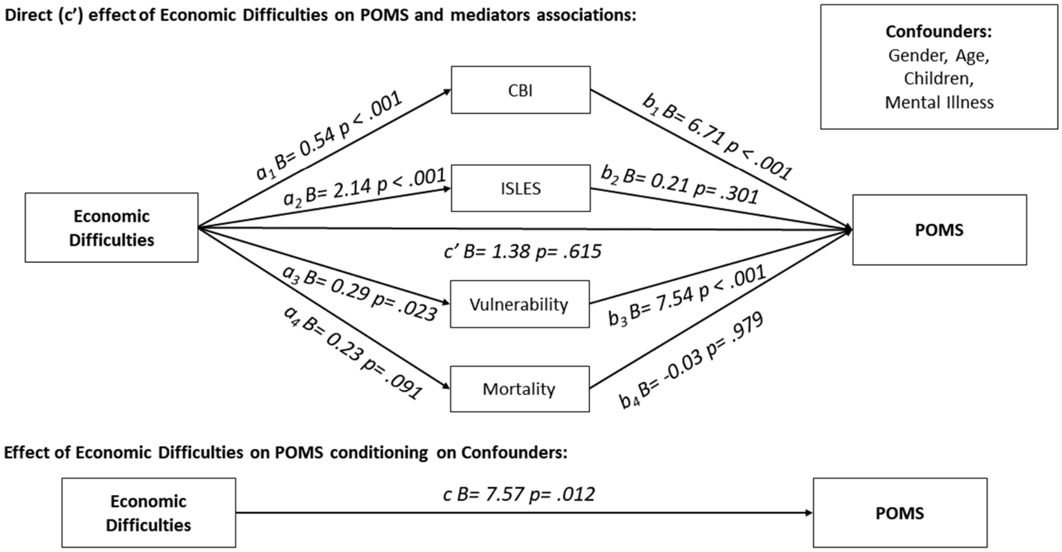

Figure S8. Mediation of the effect of Economic difficulties on POMS.

Predictor: Working from Home

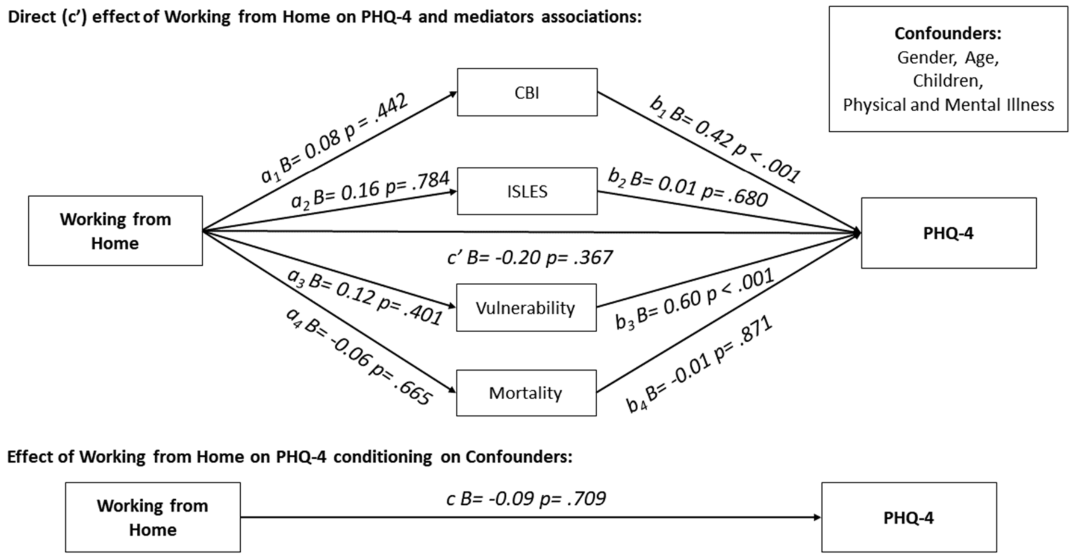

Figure S9. Mediation of the effect of Working from home on PHQ-4.

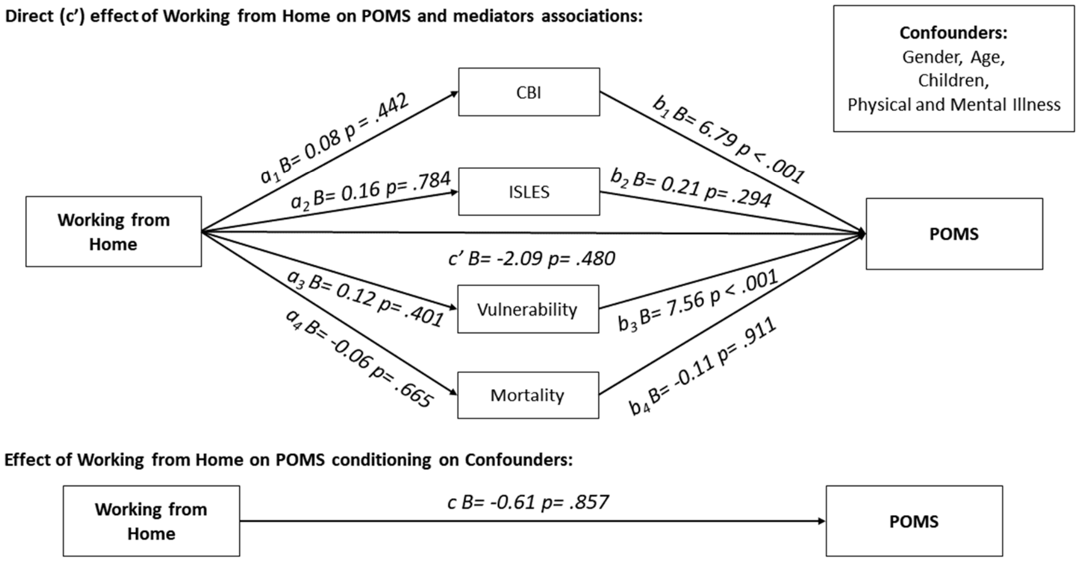

Figure S10. Mediation of the effect of Working from home on POMS.

Predictor: Leaving Home to Work

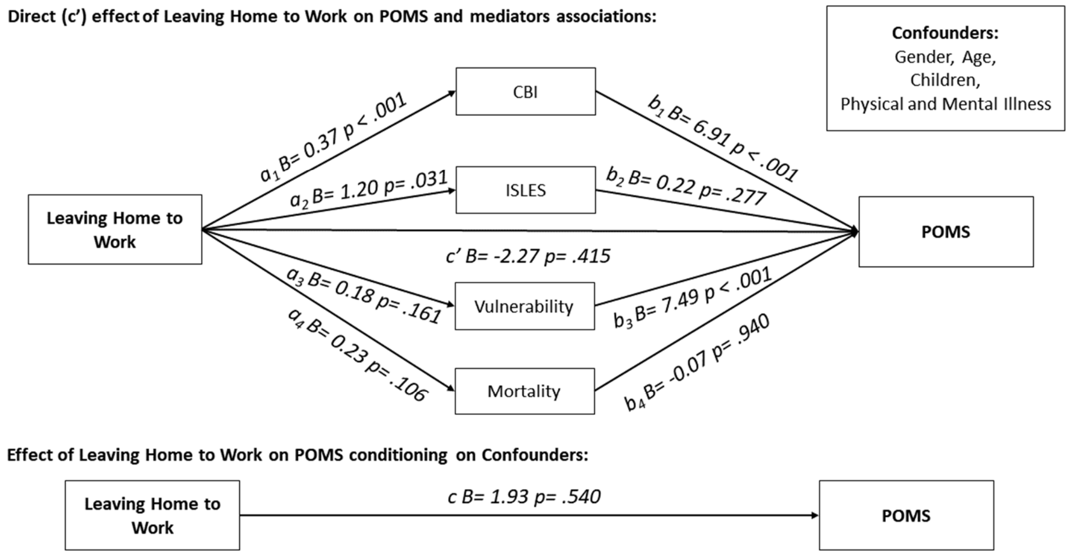

Figure S11. Mediation of the effect of Leaving home to work on POMS.

Predictor: COVID-19 Deaths

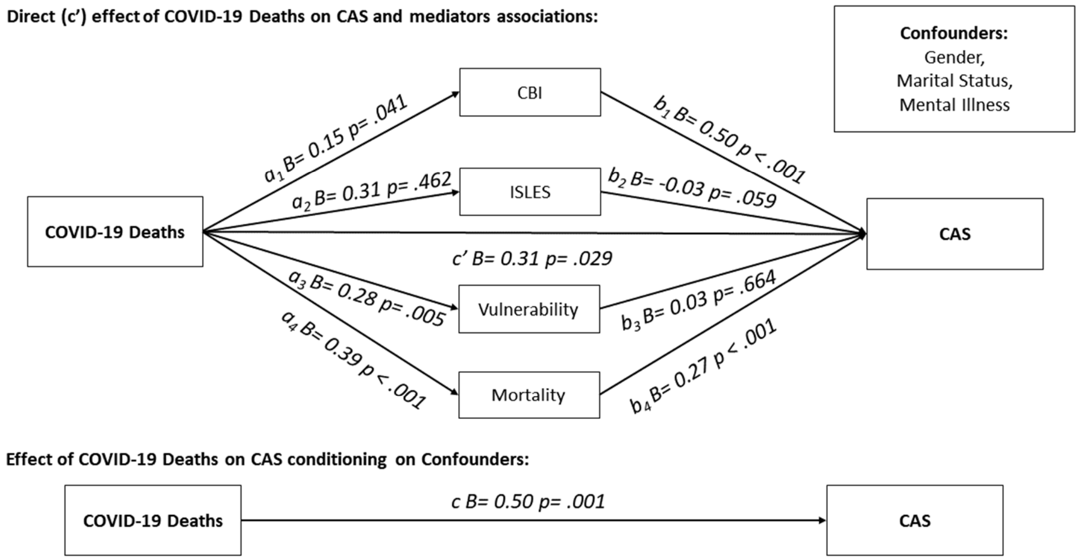

Figure S12. Mediation of the effect of COVID-19 Deaths on CAS.

3. Replication of Milman et al. (2020) Mediation models

Table S1. Testing the mediation hypothesis with Core Beliefs Violation as the mediator.

	Outcome: Core Beliefs Violation				Outcome: Personal Health Questionnaire-4				Outcome: COVID Anxiety Scale			
	Pr	B	SE	*p*	Pr	B	SE	*p*	Pr	B	SE	*p*
COVID diagnosis	***a***	**0.43**	**.15**	**.003**	*c′*	0.07	.32	.835	*c′*	0.09	.29	.759
CBI					***b***	**1.09**	**.08**	**<.001**	***b***	**0.86**	**.08**	**<.001**
COVID death	***a***	**0.18**	**.08**	**.023**	*c′*	0.14	.17	.419	***c′***	**0.42**	**.15**	**.005**
CBI					***b***	**1.09**	**.08**	**<.001**	***b***	**0.85**	**.07**	**<.001**
Job loss or reduction	***a***	**0.57**	**.09**	**<.001**	*c′*	-0.25	.22	.250	*c′*	0.12	.20	.532
CBI					***b***	**1.11**	**.09**	**<.001**	***b***	**0.85**	**.08**	**<.001**
Economic difficulties	***a***	**0.61**	**.09**	**<.001**	*c′*	-0.06	.22	.801	*c′*	0.14	.20	.475
CBI					***b***	**1.10**	**.09**	**<.001**	***b***	**0.85**	**.08**	**<.001**
Childcare loss	***a***	**0.38**	**.10**	**<.001**	*c′*	0.03	.21	.901	*c′*	0.22	.19	.250
CBI					***b***	**1.09**	**.08**	**<.001**	***b***	**0.85**	**.08**	**<.001**

Note. Pr = Parameters, *a* = effect of exposure on mediator, c′ = controlled direct effect of exposure on outcome, *b* = effect of mediator on outcome, controlling for exposure. B = Regression coefficient for each parameter, SE = standard error of regression coefficient. CBI = Core Beliefs violation Inventory. Significant effects highlighted in bold.

Table S2. Testing the mediation hypothesis with Meaning Making as the mediator.

	Outcome: Meaning Making				Outcome: COVID Anxiety Scale				Outcome: Personal Health Questionnaire-4			
	Pr	B	SE	*p*	Pr	B	SE	*p*	Pr	B	SE	*p*
COVID diagnosis	*a*	.21	.8	.793	*c′*	.55	.35	.12	*c′*	.47	.31	.124
ISLES-SF					***b***	**-.08**	**.02**	**<.001**	***b***	**-.07**	**.01**	**<.001**
COVID death	*a*	.28	.42	.505	*c′*	.35	.19	.061	***c′***	**.59**	**.16**	**<.001**
ISLES-SF					***b***	**-.08**	**.02**	**<.001**	***b***	**-.08**	**.01**	**<.001**
Job loss or reduction	***a***	**-2.55**	**.52**	**<.001**	*c′*	.2	.24	.394	***c′***	**.44**	**.21**	**.035**
ISLES-SF					***b***	**-.07**	**.02**	**<.001**	***b***	**-.07**	**.02**	**<.001**
Economic difficulties	***a***	**-2.3**	**.52**	**<.001**	*c′*	.45	.24	.057	***c′***	**.5**	**.21**	**.016**
ISLES-SF					***b***	**-.07**	**.02**	**<.001**	***b***	**-.07**	**.02**	**<.001**
Childcare loss	***a***	**-2.58**	**.52**	**<.001**	*c′*	.26	.24	.273	*c′*	.37	.21	.075
ISLES-SF					***b***	**-.07**	**.02**	**<.001**	***b***	**-.07**	**.02**	**<.001**

Note. Pr = Parameters, *a* = effect of exposure on mediator, c′ = controlled direct effect of exposure on outcome, *b* = effect of mediator on outcome, controlling for exposure. B = Regression coefficient for each parameter, SE = standard error of regression coefficient. ISLES-SF = Integration of Stressful Life Experiences Scale – Short Form. Significant effects highlighted in bold.
